# Adverse orienting effects on visual working memory encoding and maintenance

**DOI:** 10.3758/s13423-016-1205-4

**Published:** 2016-11-28

**Authors:** Benchi Wang, Chuyao Yan, Zhiguo Wang, Christian N. L. Olivers, Jan Theeuwes

**Affiliations:** 1 Department of Experimental and Applied Psychology, Vrije Universiteit Amsterdam, Van der Boechorststraat 1, 1081 BT Amsterdam, The Netherlands; 2 Department of Psychology, Hangzhou Normal University, Hangzhou, China; 3 Department of Cognitive Science, Macquarie University, Sydney, Australia

**Keywords:** Visual working memory, Inhibition of return, Pre-cue, Retro-cue

## Abstract

Visual working memory (VWM) representations can be strengthened by pre-cues presented before, and retro-cues presented after, the memory display, providing evidence that attentional orienting plays a role in memory encoding and maintenance. It is unknown whether attentional orienting to VWM stimuli can also have adverse effects (known as *inhibition of return*; IOR), as has been found for perceptual-cueing tasks. If so, this would provide further evidence for common attentional orienting mechanisms for mnemonic and perceptual representations. In Experiment 1, we used pre-cueing and demonstrated an increased encoding probability, but not precision, at short SOAs, but probability decreased at long SOAs, reminiscent of the classic IOR findings. In Experiment 2, we used retro-cueing and showed that it improved memory performance, unless attention was cued back to the center of the display by a second cue. In this case, the deleterious effects were on precision, indicating that the item was still retained, but its quality of representation suffered. Together, these results provide further evidence for universal spatial attentional mechanisms operating on perceptual as well as mnemonic representations.

**Electronic supplementary material:**

The online version of this article (doi:10.3758/s13423-016-1205-4) contains supplementary material, which is available to authorized users.

Visual working memory (VWM) is a limited-capacity system that serves to temporally maintain visual information and is essential for many ongoing cognitive tasks. The role of attention in VWM is undisputed: It affects encoding into VWM (Schmidt, Vogel, Woodman, & Luck, 2002), is used to retrieve information from VWM (Theeuwes, Kramer, & Irwin, 2011), and plays a crucial role in the selective maintenance of items within VWM (Vogel, Woodman, & Luck, 2005). There are several ways to orient attention to specific items in memory. For example, when a pre-cue is presented before the memory array, attention focuses onto its spatial location, and items presented at that location are more likely to be transferred into VWM (Awh & Pashler, 2000; Makovski & Jiang, 2007; Schmidt et al., 2002; Vogel et al., 2005). Alternatively, cues can be presented after the memory array has been switched off, allowing the orientation of attention to a spatial location within an internal memory representation (Delvenne, Cleeremans, & Laloyaux, 2009; Griffin & Nobre, 2003; Makovski & Jiang, 2007; Murray, Nobre, Clark, Cravo, & Stokes, 2013; van Moorselaar, Gunseli, Theeuwes, & Olivers, 2015; van Moorselaar, Olivers, Theeuwes, Lamme, & Sligte, 2015). Such so-called *retro-cues* improve the memory representation of the cued item.

In perceptual attention research, there is also a long research tradition investigating *adverse* effects of attention-directing cues on perceptual processing (Klein, 2000; Posner & Cohen, 1984; Wang, Hilchey, Cao, & Wang, 2014). This type of research uses a typical task, in which a cue appears to either the left or the right of fixation. Typically, participants are faster and more accurate in responding to targets that appear at the cued than at the uncued location, referred to as the *cue facilitation effect* (Posner, 1980). Notably, when there is a delay between the offset of the cue and the onset of the target, participants are *slower* in responding to targets at the cued than at the uncued location, an effect called *inhibition of return* (IOR; cf. Posner & Cohen, 1984). Thus, orienting in the outside world shows a typical biphasic pattern in which attentional facilitation is followed by inhibition. It has been argued that the mechanism responsible for IOR serves a novelty-seeking, foraging function by inhibiting attention from returning to previously examined locations (Klein, 2000).

Previous studies have shown that IOR is mediated by spatial working memory (Castel, Pratt, & Craik, 2003). Here we report two experiments investigating the reverse: the extents to which IOR modulates VWM encoding (Exp. 1, using a pre-cueing procedure) and maintenance (Exp. 2, using a retro-cueing procedure).

## Experiment 1

In Experiment 1, we tested whether IOR-like effects occurred on encoding into VWM. We first presented a spatial cue that was either valid or invalid. The SOA between the cue and the memory display was either short (200 ms) or long (400 ms). Previous studies investigating IOR on perceptual processing have shown that with these SOAs, one should observe attentional facilitation followed by inhibition (see Klein, 2000, for a review). We tested whether the same biphasic effect seen in perceptual processing can be found on VWM performance. More specifically, we investigated whether IOR affected the *probability* of encoding, or the *precision*, by employing a mixture-modeling approach (Bays, Catalao, & Husain, 2009).

### Method

#### Participants

Sixteen adults (11 females, 5 males; mean age = 22.8 years) took part for money compensation or course credits. They all provided written informed consent, and all reported normal color vision and normal or corrected-to-normal visual acuity. Eight participants were run in China, and eight were run in the Netherlands.

#### Apparatus

An HP Compaq 8000 Elite computer with a 21-in. color monitor controlled the timing of the events and generated stimuli on a gray screen (17 cd/m^2^ in China, and 19 cd/m^2^ in the Netherlands). A CRT monitor was used in China, and an LCD monitor was used in the Netherlands. Stimulus presentation and response registration were controlled by custom scripts written in Python. Participants were tested in a dimly lit laboratory, and they held their head on a chinrest located 71 cm away from the monitor. The research protocol was approved by the Scientific and Ethical Review Committee of the Faculty of Psychology and Education of VU University and by the Institutional Review Board of the Center for Cognition and Brain Disorders, Hangzhou Normal University.

#### Stimuli

**Fig. 1:**
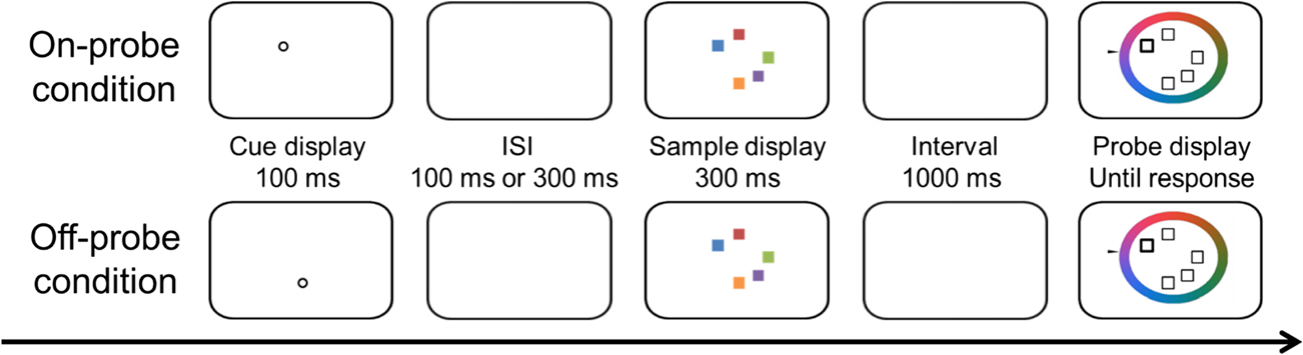
The procedure of Experiment 1. A pre-cue was shown for 100 ms before the memory display (300 ms), and the interstimuli interval (ISI) could be 100 and 300 ms. After a delay period (1,000 ms), a color wheel representing continuous color values and a test cue signaling the to-be-recalled item were presented. Participants recalled the feature value of the indicated item by using a mouse to select a value on the wheel

Participants were required to memorize the colors of five colored squares (0.6° × 0.6°). For each trial, the color of each square was randomly chosen from one of 12 equiluminant colors (ranges: 65–70 cd/m^2^ in China and 36–44 cd/m^2^ in the Netherlands), evenly distributed along a color circle in the CIE L*a*b* color space (centered at L = 70, a = 5, b = 0, with a radius of 60), without replacement, and their locations were randomly chosen from eight equally spaced positions along an imaginary ring with a radius of 3° (see Fig. 1).

#### Procedure and design

A small fixation point (0.67° × 0.67°) was presented throughout the trial. A white circle serving as a pre-cue (217 cd/m^2^ in China and 176 cd/m^2^ in the Netherlands) with a radius of 0.5° was presented for 100 ms. This cue appeared at the probed item’s location (on-probe condition) or at one of the nonprobed items’ locations (off-probe condition), followed by a time interval of either 100 or 300 ms, randomized within blocks. Then the memory array was presented for 300 ms. After a 1,000-ms memory delay, the probe display was presented until response, containing five empty squares (0.6° × 0.6°) and a continuous response wheel (subtending 1.5° wide, 7° radius) of 180 color segments. This display remained on until the response. A bolder square indicated which item participants had to report by selecting one of 180 values on the color wheel (randomly rotated), using the mouse. The selected color value was indicated by a small black arrowhead cursor. While the mouse was being moved, the cued square was filled with the color value matching the selected value. Accuracy was emphasized. There were 2 (Pre-Cue Location: on-probe vs. off-probe) × 2 (SOA: 200 vs. 400 ms) conditions, randomly mixed, run in two successive sessions held within 5 days of each other. Each condition contained 200 trials, for a total of 800 trials. The experiment was preceded by 20 practice trials. To maximize the number of trials for each condition, the validity of the pre-cue was 50%, although this might in fact be suboptimal for observing inhibition.

#### Analysis

Response error was calculated by subtracting each probed item’s correct value from the response value. A mixture-modeling analysis (Bays, Catalao, & Husain, 2009) was conducted on these response error data (see the supplementary material, Figs. S1 and S2, for a histogram of the response error data across all participants), to derive the parameters of the response error distribution. This distribution was assumed to consist of a uniform distribution of response errors (for guessing trials), one von Mises (circular normal) distribution of response errors for nonguessing trials, and four von Mises distributions of response errors for memory substitutions, in which observer reported one of the other items in the display. By using maximum likelihood estimation, the distribution of the response error data from each condition was entered into the model

$$ p(e)=\left(1-g-\beta \right){\phi}_{\sigma }(e)+g\frac{1}{2\pi }+\beta \frac{1}{m}{\displaystyle \sum_1^m{\phi}_{\sigma }}\left({e}_m\right), $$

where two input parameters *e* (response errors) and *m* (the number of nontarget items) are required, and three output parameters *g* (guess rate; i.e., the proportion of the guess trials), *β* (swap errors; i.e., the proportion of misremembering trials), and *σ* (standard deviation; i.e., the width of the mixture distribution, reflecting the precision of the memory representation) will be given. We used the MemToolbox (a MATLAB toolbox; Suchow, Brady, Fougnie, & Alvarez, 2013) to fit the mixture distribution of the response errors. We also fitted the two-parameter model of Zhang and Luck (2008), which yielded the same pattern of results (see the supplementary material).

### Results

The response error distribution and its model-fitted results (see the supplementary material, Fig. S1) for each condition were derived from the best-fitting parameter values of the overall trials. The guess rate (*g*), swap error (*β*), and standard deviation (*σ*) were each entered in a repeated measures analysis of variance (ANOVA) with Pre-Cue Location (on-probe vs. off-probe) and SOA (200 vs. 400 ms) as factors.

**Fig. 2:**
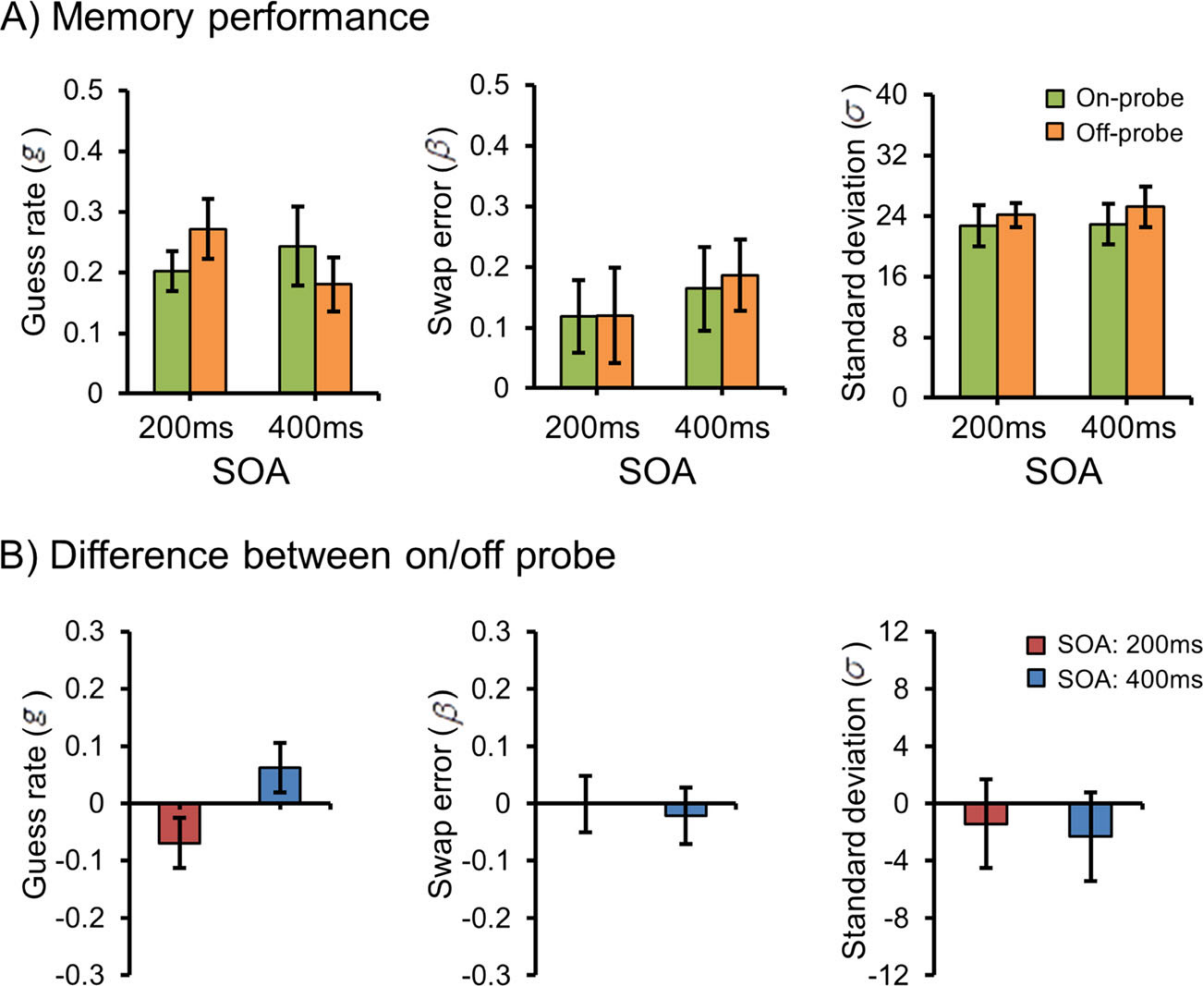
The results of Experiment 1. (**a**) Guess rates, swap errors, and standard deviations of each condition. (**b**) Differences between the *on*- and *off-probe* conditions in terms of guess rates, swap errors, and standard deviations. Error bars denote within-subjects 95% confidence intervals (Morey, 2008)

Figure 2a (left panel) presents the means of the guess rates for all conditions, and Fig. 2b (left panel) presents the differences in guess rates between the different pre-cue locations. No significant main effect was observed for pre-cue location, *F* < 1, *p* > .9, although there was a tendency toward fewer guesses with increasing SOA, though this was not significant, *F*(1, 15) = 3.1, *p* = .1, *η*_p_^2^ = .17. Importantly, however, we observed a significant interaction, *F*(1, 15) = 18.5, *p* = .001, *η*_p_^2^ = .55. Planned comparisons showed that at SOA 200 ms, the guess rate was marginally lower for on- than for off-probe conditions, *t*(15) = 1.89, *p* = .079, an effect that was reversed in the SOA 400-ms condition, in which the guess rate was now higher for on- than for off-probe performance, *t*(15) = 2.4, *p* = .03.

The middle and right panels of Fig. 2a present the means of swap errors and standard deviations, respectively, for all conditions, and the same panels in Fig. 2b present the differences in swap errors and standard deviations, respectively, between the different pre-cue locations. No significant main effects or interactions were observed for either of these measures, all *F*s < 2.1, all *p*s > .17.

### Discussion

The present experiment shows the classic biphasic effect of pre-cues that is reminiscent of IOR, but now on VWM performance, with early facilitation at the short SOA followed by inhibition at the long SOA. Furthermore, this effect was found for memory probability (reflected by the guess rate), but not for memory precision (reflected by the standard deviation). We propose that at the long SOA, due to IOR, the (pre-cued) location became inhibited before the sample display was presented. Once the display was presented, IOR at that location reduced the probability that the stimulus presented at that location would be encoded into VWM. However, if it *was* encoded, it was represented with the same precision as when it was attended. This suggests an all-or-none mechanism of IOR, in which on some trials attention was still there (with full precision as a consequence), whereas on other trials it was withdrawn (and the item was not even encoded).

## Experiment 2

In Experiment 2, we employed retro-cuing in which the cue was presented *after* extinguishing the memory array. Following the presentation of a retro-cue, it is likely that attention remains focused on the location of the retro-cue unless we force attention to move elsewhere. To reorient attention away from the cued location, we used a cued-back condition (as was used in the classic Posner & Cohen [1984] IOR paradigm) to promote the reorientation of attention away from the cued item, and thereby inhibit the return of attention to that item. In Experiment 2, in one condition, we presented a second cue in the center of the display to draw attention back to the center. Because the mechanisms underlying IOR bias responses against previously attended locations, the location of the retro-cue should become suppressed when attention is drawn back to the center.

### Method

**Fig. 3:**
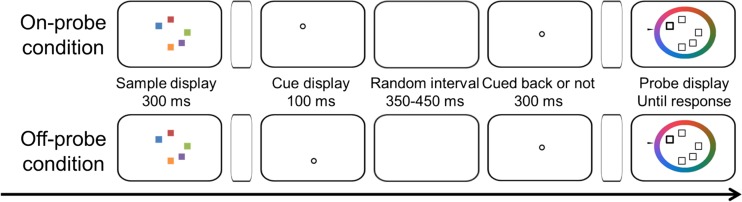
The procedure of Experiment 2. First, the memory display was shown for 300 ms, and then the retro-cue was shown for 100 ms, after an interval of 200 ms. A random interval (350–450 ms) followed the retro-cue, and then another cue or no cue. Finally, a color wheel representing continuous color values and a test cue signaling the to-be-recalled item were presented. Participants recalled the feature value of this item by using a mouse to select a value on the wheel

Thirty-one adults (18 females, 13 males; mean age 23.9 years) took part for money compensation or course credits. The procedure was similar to that of Experiment 1, except that instead of a pre-cue, a retro-cue was presented 200 ms after the offset of the memory display. The retro-cue was shown for 100 ms, followed by a random retention interval (350–450 ms). Then, depending on the condition, either no second cue was presented (not-cued-back condition) or a second cue was presented for 300 ms at the center of the screen (cued-back condition); see Fig. 3 for an example. Each participant completed 20 practice trials and 1,000 experimental trials, with 2 (Retro-Cue Location: on-probe vs. off-probe) × 2 (Attention Shift: cued back vs. not cued back) conditions randomly mixed. The validity of the retro-cue (i.e., the proportion of the on-probe condition) was 20%, to make sure each to-be-memorized item had an equally cued probability, so that each on-probe condition contained 100 trials and each off-probe condition contained 400 trials. In all other respects, including the data analysis, this experiment was the same as Experiment 1.

### Results

The response error distribution and its model-fitted results for each condition are illustrated in the supplementary material, Fig. S2. As in Experiment 1, repeated measures ANOVAs were conducted on the guess rates (*g*), swap errors (*β*), and standard deviations (*σ*), separately, with the variables Retro-Cue Location (on-probe vs. off-probe) and Attentional Shift (cued back vs. not cued back).

**Fig. 4:**
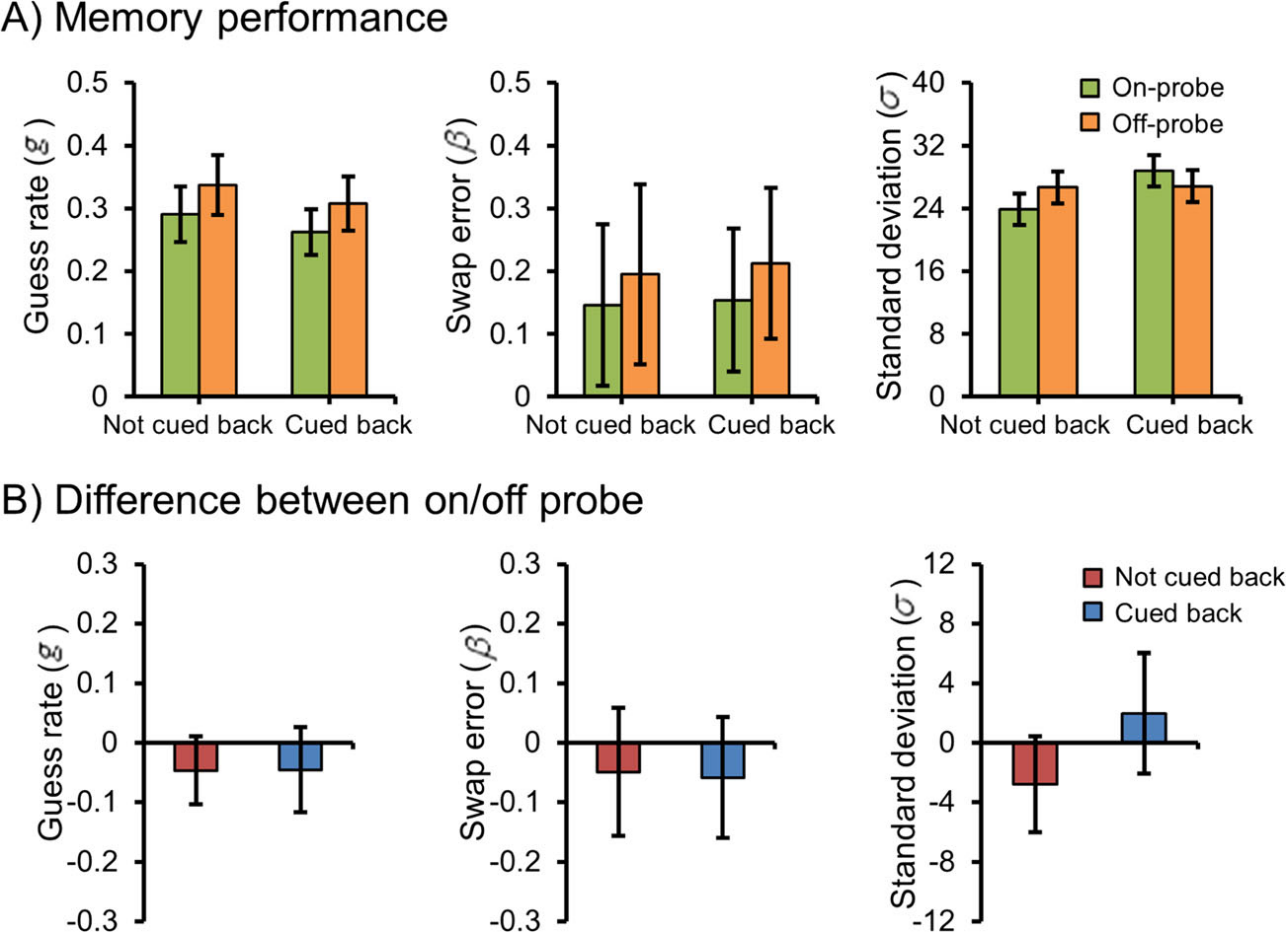
The results of Experiment 2. (**a**) Guess rates, swap errors, and standard deviations of each condition. (**b**) Differences between the *on-* and *off-probe* conditions in terms of guess rates, swap errors, and standard deviations. Error bars denote within-subjects 95% confidence intervals

The means of the guess rates for all conditions are presented in Fig. 4a (left panel), and the differences in guess rates between the on-probe and off-probe conditions are presented in Fig. 4b (left panel). We found a main effect of retro-cue location, *F*(1, 30) = 8.94, *p* = .006, *η*_p_^2^ = .23, but not of attention shift, *F*(1, 30) = 2.04, *p* = .163, *η*_p_^2^ = .06. There was no two-way interaction, *F*(1, 30) = 0.001, *p* = .979, *η*_p_^2^ < .001. These data indicate a reliable retro-cue benefit, since the guess rate was lower in the on-probe than in the off-probe condition, no matter whether or not attention was shifted to the center.

Figure 4a (middle panel) shows the mean swap errors for all conditions, and the differences in swap errors between the on-probe and off-probe conditions are shown in Fig. 4b (middle panel). No significant main effects or interactions were observed, all *F*s < 2.4, all *p*s > .132.

Finally, Fig. 4a (right panel) shows the means of the standard deviations for all conditions. Figure 4b (right panel) shows the differences in standard deviations between the on-probe and off-probe conditions. We observed a significant main effect of attention shift, *F*(1, 30) = 6.37, *p* = .015, *η*_p_^2^ = .18, but not of retro-cue location (on- and off-probe condition), *F*(1, 30) = 0.16, *p* = .688, *η*_p_^2^ = .01. Importantly, there was a reliable two-way interaction, *F*(1, 30) = 9.44, *p* = .004, *η*_p_^2^ = .24. Planned comparisons indicated that in the on-probe condition, the standard deviation was lower than in the off-probe condition when there was no cue back, *t*(30) = 2.46, *p* = .02, but that this decreased standard deviation was no longer present when the cue back was delivered, *t*(30) = 1.38, *p* = .178.

### Discussion

Consistent with previous studies, a clear retro-cue benefit was observed for both memory probability (in terms of guess rate) and memory precision (in terms of standard deviation). When the retro-cue was valid, the memory probability of the cued item was significantly increased in both the cued-back condition and the not-cued-back condition, showing that attention was directed to the cued item, at the expense of the noncued items. More importantly, although the benefit for probability was retained, the retro-cue benefit for memory precision was no longer present in the cued-back condition relative to the condition in which there was no second cue (the not-cued-back condition), resulting in a cue by probe location interaction. Thus, memory precision was relatively suppressed, consistent with a IOR-like mechanism.

Note that in the cued-back condition, the on-probe location was no worse than the off-probe location. One may argue if this on-probe item had been inhibited (as a mechanism such as IOR would imply) because attention was pulled away by the second cue, one would have expected worse performance in this condition (the on-probe cued-back condition) than in the off-probe (cued-back) condition. However we argue that memory, unlike attention, serves to create a *sustained* representation. By cueing an item, it is consolidated for later report, as is evident from the fact that probability of report improved reliably for cued items—regardless of whether or not attention was then cued away again. This means that any inhibitory attention-related effects can only start to operate *after*—and thus will be expressed *relative to*—a sustained benefit. Interestingly, our data are consistent with such mechanisms: We did not see an effect of the second cue on encoding probability (because the first cue ensured that the item was already consolidated in memory, even when attention was later cued back), but instead an effect on memory precision. This dissociation between probability and precision also explains why we came to a different conclusion than Hollingworth and Maxcey-Richard (2013), who claimed that VWM maintenance should be dissociated from the locus of visual attention. In their experiment, they found that attention shifts during the delay period had no impact on retro-cue benefits. However, they did not include memory precision as a measure, only the probability of correct report. As we showed here, cueing attention away during the delay period may indeed not affect the probability of reporting an item, only its precision.

## General discussion

The present study reveals adverse mechanisms of spatial orienting on VWM performance that are reminiscent of IOR observed for perceptual presentations. In Experiment 1 we used a pre-cue to orient attention to specific items in the memory array and revealed a biphasic effect on the probability of encoding the item into VWM, with first facilitation and then inhibition. Previous studies have shown a facilitation effect of pre-cues on memory performance (e.g., Awh & Pashler, 2000); the present study is the first to show that such cues also have adverse effects on VWM performance. For Experiment 2 we used a retro-cue technique, and consistent with previous studies, memory performance benefits (memory probability in the present study) appeared when the item that was retro-cued needed to be recalled (on-probe) versus the condition in which another item needed to be recalled (off-probe; see, e.g., Griffin & Nobre, 2003). Crucially, however, when attention was pulled back to the middle of the screen, the retro-cue benefit on memory precision waned, whereas the benefits on probability remained. This suggests a suppression of memory precision when attention was withdrawn, an effect similar to the occurrence of IOR in Posner-like attentional-cueing tasks.

The present findings are consistent with a large functional overlap between attentional and VWM mechanisms (Awh & Jonides, 2001; Chun, 2011; Kiyonaga & Egner, 2012, 2016; Olivers, 2008; Peters, Kaiser, Rahm, & Bledowski, 2015; Sahan, Verguts, Boehler, Pourtois, & Fias, 2015; Theeuwes, Belopolsky, & Olivers, 2009). Although IOR is functional when searching for an object in the outside world (facilitation of foraging; Klein, 2000), it may be less useful when holding items in VWM. In addition to the role of IOR as foraging facilitator in visual search, recent studies have stressed the effect of IOR on motoric/decision-making processes (so-called *output-based IOR*; see Hilchey, Klein, & Satel, 2014), especially when participants make saccadic eye movements. Even though we did not monitor eye movements, in the present study it is highly unlikely that eye movements were made, because participants were instructed to memorize the colors of five squares presented on an imaginary circle around fixation. If anything, the best strategy to memorize the colors was to remain fixated on the center. Furthermore, it is unclear how motor inhibition would explain a reduced probability of encoding (Exp. 1) or memory precision (Exp. 2).

The present study shows that pre-cues led to reduced memory probability (at a long SOA), whereas retro-cues led to reduced memory precision (when attention was withdrawn). It is likely that this difference relates to a fundamental difference in the information that was available at the time of cueing. At the moment of a pre-cue, by definition, no item information is yet available, only location information. Attending to that location will thus help reduce the effective set size (at the expense of the other items) and increase the likelihood of encoding the cued item. In the retro-cue condition, however, the item has already been encoded (at least with high likelihood). Given this benefit, there is no incentive to drop it. All that can then be affected by an inhibitory mechanism is the precision of the memory. Thus, we suggest that the attentional mechanisms of pre- and retro-cueing may be the same, including IOR, but that the effects may differ, depending on whether the to-be-remembered item is already available or still has to be encoded.

## Electronic supplementary material

Below is the link to the electronic supplementary material.

ESM 1 (DOCX 1.31 mb)
