# Supplementary material for: Adverse orienting effects on visual working memory encoding and maintenance
Source: Psychon Bull Rev. 2016 Nov 28;24(4):1261–7. doi: 10.3758/s13423-016-1205-4 (PMC5570809; doi:10.3758/s13423-016-1205-4)
Supplement: Supplementary file 1 — (DOCX 1.31 mb) [file 13423_2016_1205_MOESM1_ESM.docx]

**Supplementary Material**

“ Adverse orienting effects on visual working memory encoding and maintenance
” by Benchi Wang, Chuyao Yan, Zhiguo Wang, Christian N. L. Olivers, and Jan Theeuwes.

Supplementary figures


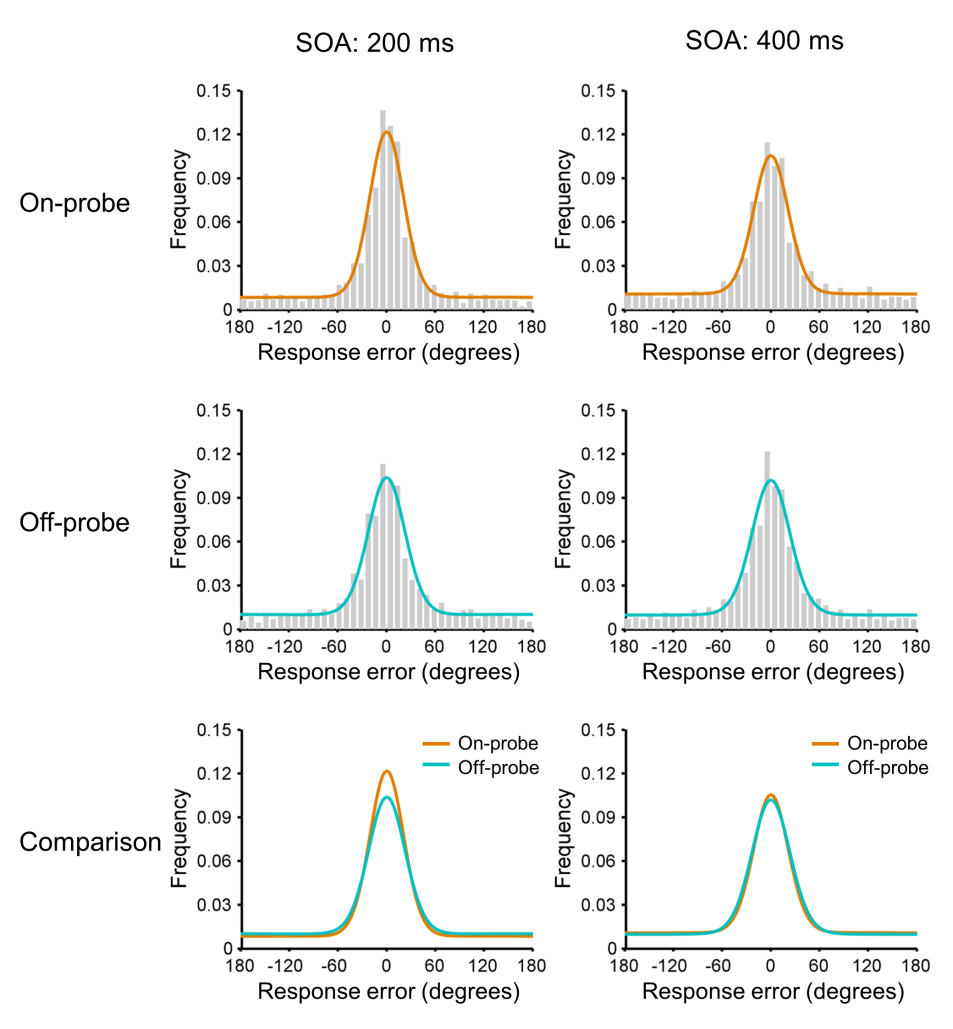


***Figure S1****.* Response error histograms across all participants in Experiment 1. Lines with different colors indicate the best fitting mixture model (i.e., the swap model by Bays, Catalao, & Husain, 2009) for each condition.


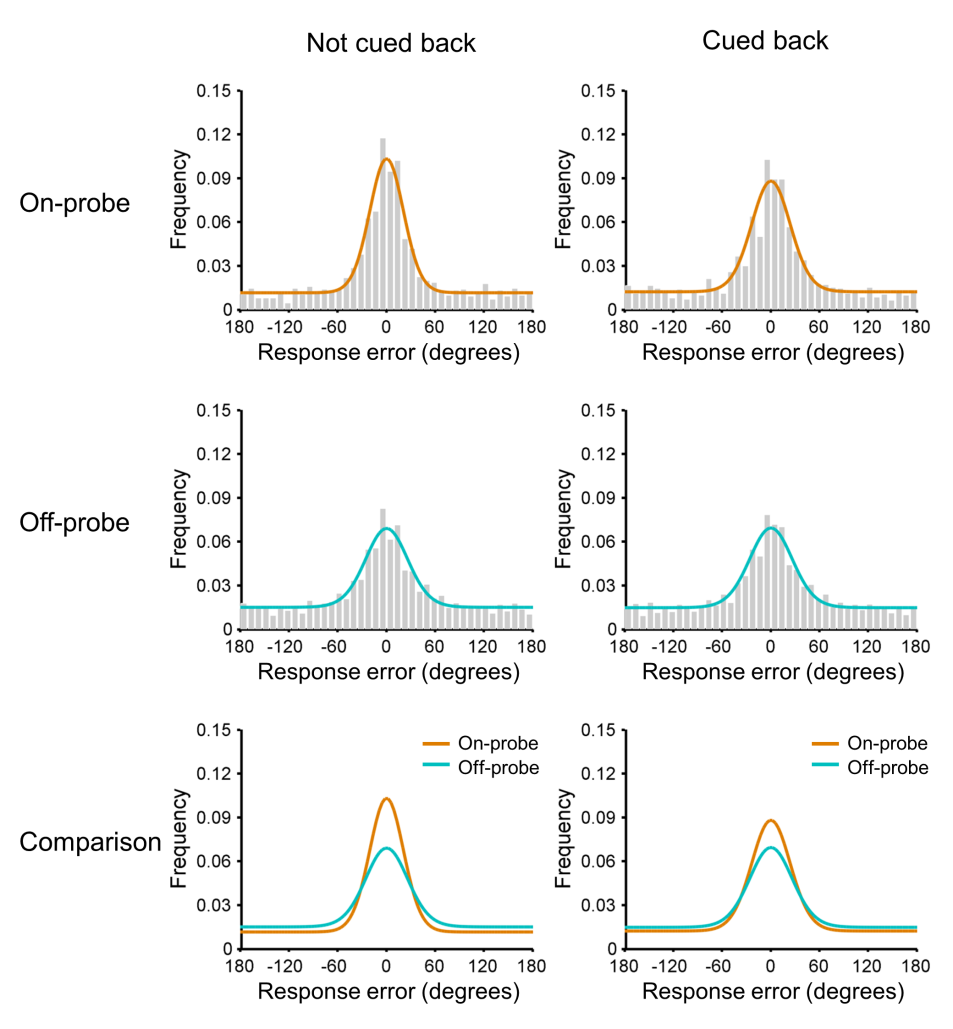


***Figure S2.*** Response error histograms across all participants in Experiment 2. Lines with different colors indicate the best fitting mixture model for each condition.

Supplementary results

In addition to the mixture modeling analysis from Bays, Catalao, and Husain (2009), we also fitted the model from Zhang and Luck (2008). In this model, the distribution only consisted of a uniform distribution of response errors (for guessing trials) and a von Mises (circular normal) distributions of response errors (for non-guessing trials). By using maximum likelihood estimation, the distribution of response errors from each condition were fitted to the following model:

$p(e)=(1-g)\varphi_{\sigma}(e)+g\frac{1}{2\pi}$,

where one input parameter $e$ (response errors) is required, and two output parameters $g$ (guess rate; i.e., the proportion of the guess trials) and $\sigma$ (standard deviation; i.e., the width of the mixture distribution, reflecting the precision of the memory representation) will be given.

Experiment 1

The response error distributions and model-fitting results of all condition are presented in Figure S3. Repeated measures ANOVAs were separately performed on guess rates ($g$) and standard deviations ($\sigma$), with variables Pre-Cue Location (on-probe vs. off-probe) and SOA (200 ms vs. 400 ms).

Figure S4A (left panel) presents the mean guess rates of all conditions, and Figure S4B (left panel) presents the guess rate difference between on- and off-probe conditions. No main effect was observed for pre-cue location, F(1, 15) = 0.85, p = .37, partial η² = .05, and SOA, F(1, 15) = 0.88, p = .364, partial η² = .06. However, a significant two-way interaction was observed, F(1, 15) = 6.94, p = .019, partial η² = .32. Planned comparisons revealed that at SOA 200 ms, guess rate was lower for on- than for off-probe conditions, t(15) = 2.13, p = .05; an effect that was reversed in the SOA 400 ms condition, where guess rate was higher for the on-probe condition, t(15) = 2.56, p = .022.

Figure S4A (right panel) presents the standard deviations of each condition, and Figure S4B (right panel) presents standard deviation difference between on- and off-probe conditions. No main effect or interaction was observed for either of these measures, all Fs < 2.79, all ps > .115.

Experiment 2

The data from two subjects were excluded from further analysis because the guess rates were too high when we fitted the data with Zhang and Luck’s model. The response error distributions and the model-fitting results of all conditions are presented in Figure S5. Repeated measures ANOVAs were separately conducted on guess rates ($g$) and standard deviations ($\sigma$), with variables Retro-Cue Location (on-probe vs. off-probe) and Attention Shift (cued back vs. not cued back).

The mean guess rates for all conditions are presented in Figure 6A (left panel), and the guess rate differences between on- and off-probe conditions are presented in Figure 6B (left panel). Significant main effects were observed for retro-cue location, F(1, 28) = 9.54, p = .005, partial η² = .25, but not for attention shift, F(1, 28) = 2.19, p = .15, partial η² = .07. No two-way interaction was obtained, F(1, 28) = 0.69, p = .41, partial η² = .02. These results indicate a reliable retro-cue benefit as the guess rate was lower in the on-probe condition versus the off-probe condition, no matter whether attention was shifted to the center or not.

The standard deviations of each condition is presented in Figure 6A (right panel), and the standard deviation differences between on- and off-probe conditions are presented in Figure 6B (right panel). A significant main effect was observed for attention shift, F(1, 28) = 7.48, p = .011, partial η² = .21, but not for retro-cue location, F(1, 28) = 0.95, p = .338, partial η² = .03. A significant two-way interaction was obtained, F(1, 28) = 12.92, p = .001, partial η² = .32. Planned comparisons showed that standard deviation was lower in the on-probe condition when attention was not cued back, t(28) = 3.36, p = .002; this memory benefit, however, was reversed when attention was cued back, t(28) = 1.5, p = .145. As outlined in the paper, we claim that this latter effect emerged because IOR-like effect had developed at the on-probe location.


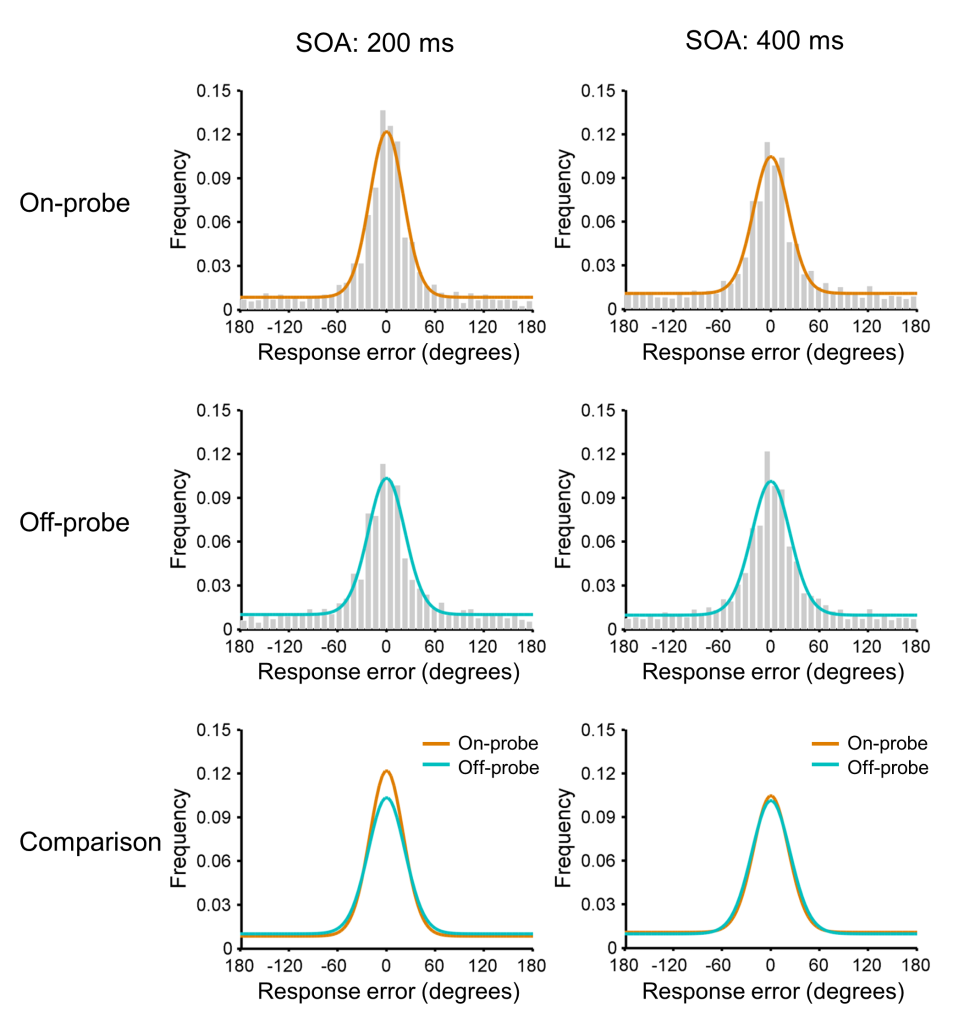


***Figure S3.*** Response error histograms across all participants in Experiment 1. Lines with different colors represent the best fitting mixture models of each condition.

*
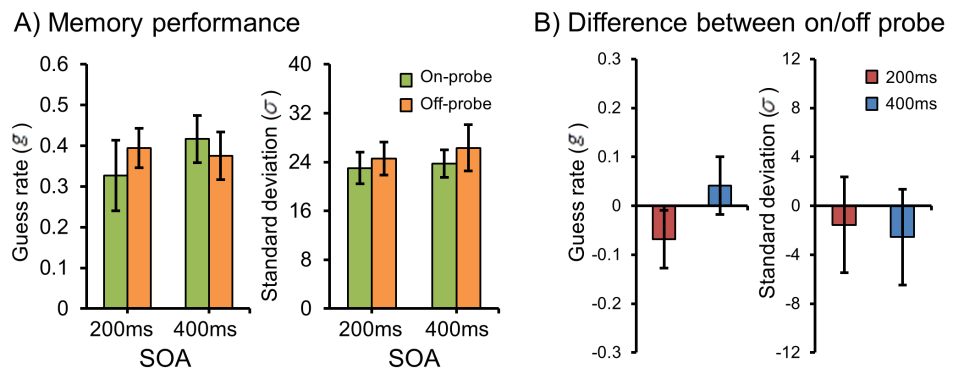
*

***Figure S4.*** The results in Experiment 1. (A) Guess rates and standard deviations of each condition. (B) The differences between *on-* and *off-probe* conditions on guess rates and standard deviations. Error bars denote within subject 95% CI.


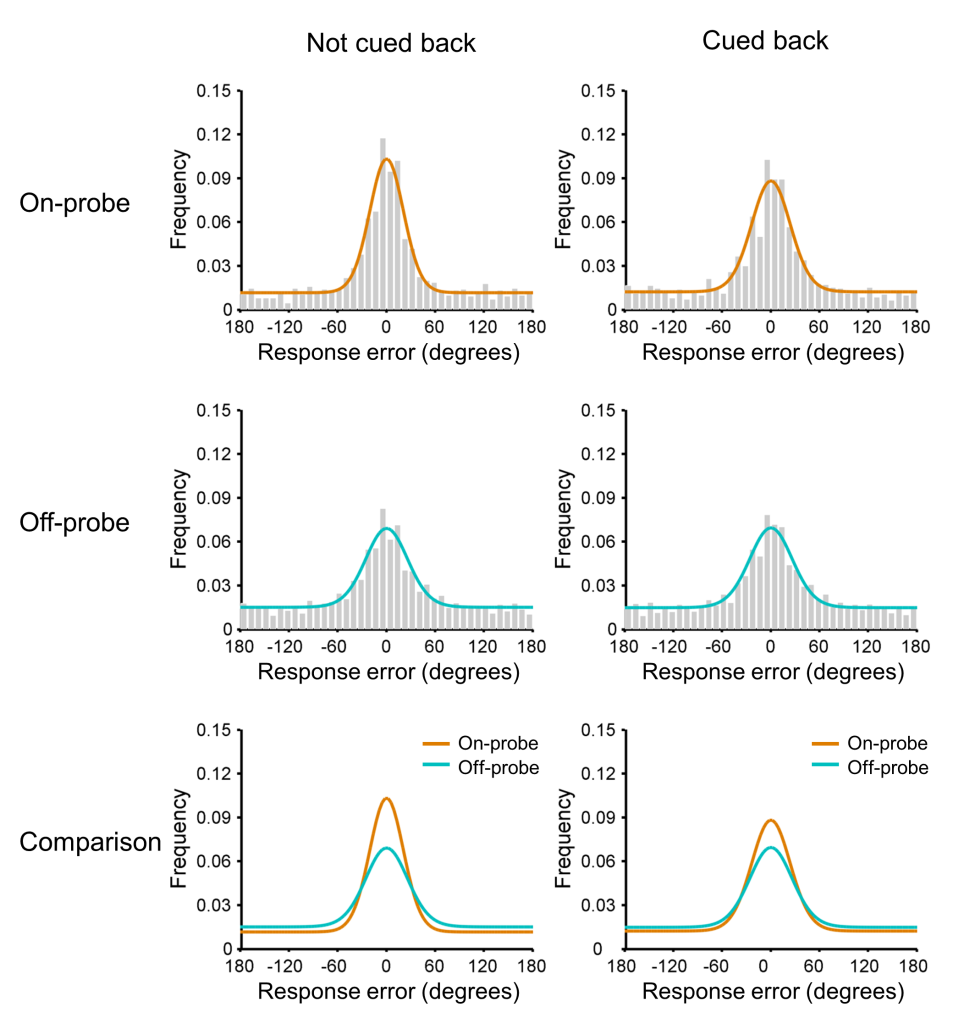


***Figure S5.*** Response error histograms across all participants in Experiment 2. Lines with different colors represent the best fitting mixture model of each condition.

*
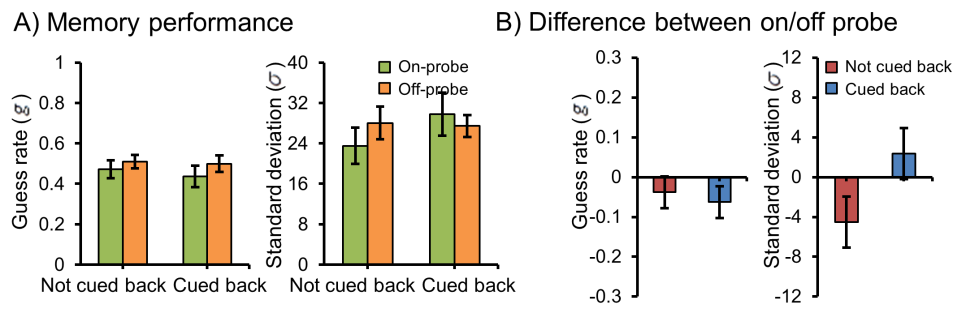
*

***Figure S6.*** The results in Experiment 2. (A) Guess rates and standard deviations of each condition. (B) The differences between *on-* and *off-probe* conditions on guess rates and standard deviations. Error bars denote within subject 95% CI.

Supplementary references

Bays, P. M., Catalao, R. F. G., & Husain, M. (2009). The precision of visual working memory is set by allocation of a shared resource. *Journal of Vision*, *9*(10), 7–7. doi:10.1167/9.10.7

Zhang, W., & Luck, S. J. (2008). Discrete fixed-resolution representations in visual working memory. *Nature*, *453*(7192), 233–235. doi:10.1038/nature06860
